# *QuickStats: *Percentage* of Employed Adults Aged ≥18 Years with Any Work-Loss Days Because of Illness or Injury in the Past 12 Months,^†^ by Sex and Age Group — National Health Interview Survey,^§^ 2017

**DOI:** 10.15585/mmwr.mm6817a7

**Published:** 2019-05-03

**Figure:**
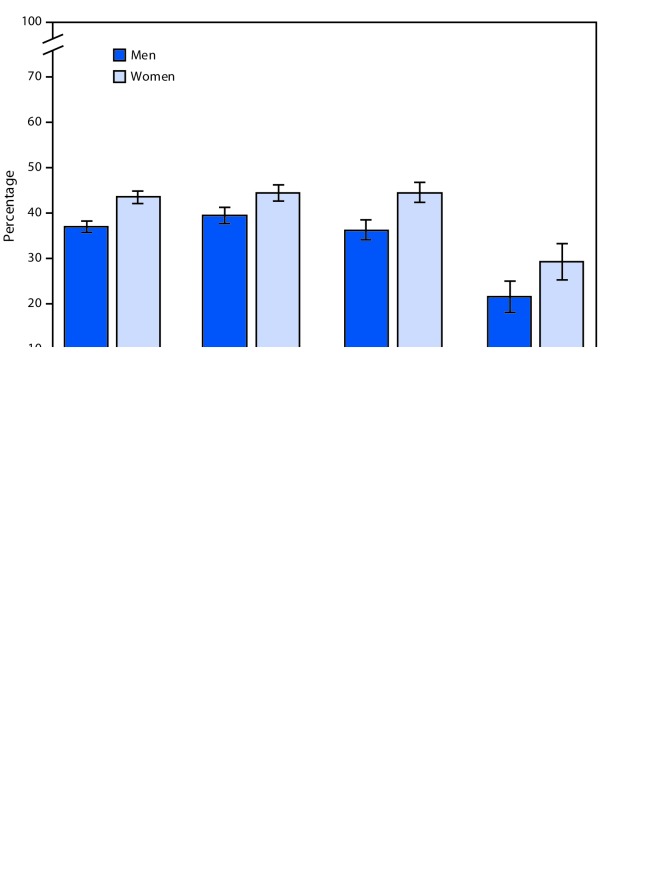
Among employed adults aged ≥18 years, women (43.5%) were more likely than men (37.0%) to have missed at least 1 day of work because of illness or injury during the past 12 months. This pattern was consistent for women and men aged 18–44 (44.5% versus 39.4%), 45–64 (44.5% versus 36.3%), and ≥65 years (29.3% versus 21.6%). Among women, having any work-loss days was similar for those aged 18–44 and 45–64 years and then declined for those aged ≥65 years. Among men, having any work-loss days decreased with age.

**Source:** National Health Interview Survey, 2017 data. https://www.cdc.gov/nchs/nhis.htm.

